# Prevalence of palatine tonsilloliths in Dominican patients of varying social classes treated in university clinics

**DOI:** 10.1038/s41598-020-58675-3

**Published:** 2020-02-03

**Authors:** J. M. Aragoneses, A. Suárez, J. Aragoneses, V. A. Brugal, M. Fernández-Domínguez

**Affiliations:** 1 Universidad Federico Henríquez y Carvajal, Santo Domingo, Dominican Republic; 2 Department of clinical dentistry, School of Biomedical Sciences, Universidad Europea de Madrid, Madrid, Spain; 3 Universidad Federico Henríquez y Carvajal, Santo Domingo, Dominican Republic; 4 Department of Paediatric dentistry and orthopedics, School of Dentistry, Universidad Autónoma de Santo Domingo, Santo Domingo, Dominican Republic; 5 Maxillofacial Surgery department at HM Hospitales. Director, Master of Oral surgery and Implants. Universidad San Pablo CEU, Madrid, Spain

**Keywords:** Epidemiology, Oral manifestations

## Abstract

The relevance of tonsils lies not only in local but also in systemic immunity. One of the most common ailments afflicting the tonsils are palatine tonsilloliths (PT), dystrophic calcifications found in the tonsillar crypts. PT prevalence reports have been conducted for Caucasian and Asian patients, but not for black patients. The aim of this cross-sectional study is to gauge the prevalence of PT in patients who sought treatment at two university clinics in the Dominican Republic, and to analyze any links with the race of patients. Two hundred and nine consecutive patients attending the dental services of two clinics located in different cities in the Dominican Republic, from March 1 to April 30, 2019, were selected. Computed tomography scans of patients were evaluated for a PT diagnosis. Determined prevalence of PT in this population sample was 5.85%. A non-significant relationship between occurrence of PT and race or kind of health service utilized was found. Nonetheless, more white patients used private health clinics while more black patients used the public health system. Previous tonsillitis was the only factor showing a significant correlation with the occurrence of PT. Also, PT prevalence was significantly higher in patients under 40 years of age. General prevalence of PT was significantly lower than reported in previous studies involving other countries/races. Considering the limitations of this study, when comparing it to a previous similar study and taking into account the Asian- and Caucasian-centric results obtained, a race influence on prevalence of calcifications may be suggested. Despite our results showing no racial differences within the Dominican Republic, black patients appear to present a lower prevalence of PT than Caucasian and Asian patients.

## Introduction

The Waldeyer’s ring is a circular structure of lymphoid tissue located in the pharynx, and its relevance lies not only in local but also in systemic immunity. Although it is diffusely present throughout the pharyngeal region, it has anatomically visible lymphatic masses called tonsils. These masses are known as the lingual, palatine, tubal and pharyngeal tonsils. The tonsils are capable of sampling the contents of the pharynx and forming antibodies against many antigens^1,2^. The largest of these masses are the two palatine tonsils. Both are individually surrounded by a capsid coated with stratified epithelium. They lie in a tonsillar cavity or depression (between the palatoglossal and palatopharyngeal folds) and have an irregular form, with multiple invaginations called crypts, within which most local immune reactions occur^3^. It is known that these lymphoid organs show specific antibodies, and T- and B-cell activity as a reaction to several antigens^4,5^.

There are several ailments involving the palatine tonsils. One of the most frequently occurring of these are tonsilloliths, also known as *tonsil concretions*, *tonsillar calculi*, *tonsil stones* and *tonsillolithiasis*: dystrophic calcifications that form in the tonsillar crypts. They can be unilateral or bilateral and, when touching the affected tonsil, a hard, submucosal mass can be felt. Small calcifications cause no symptoms; however, swelling, pain, foul taste, odor, throat irritations, otalgia and dysphagia are usually produced by larger calcifications^6,7^. Some authors have associated tonsilloliths with halitosis^8^ or even odynophagia^9^.

**Figure 1:**
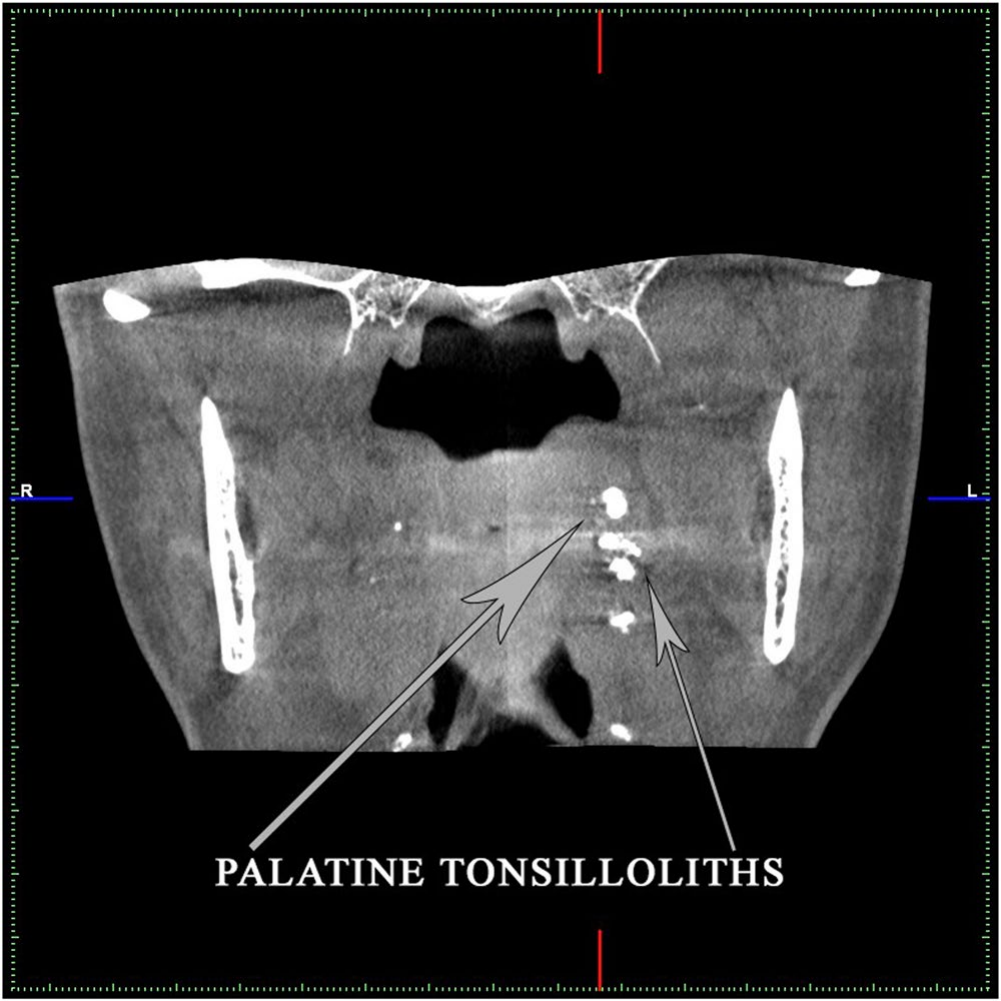
CT image of a patient diagnosed with PT. One can see multiple PT.

Palatine tonsilloliths (PT) are commonly found in patient groups ranging from 20 to 68 years, and women are determined to be more affected than men. The diagnosis is usually made based on recurrent tonsillitis and a dotted appearance with radiopaque image in the ramus zone. Radiographically, they can be viewed in the mid-portion of the mandibular ramus, where it crosses the dorsal surface of the tongue. These calcified bodies can be detected as 0.5 cm to 14.5 cm spots with a similar radiopacity to the cortical bone (more than the cancellous bone). Larger symptomatic calcifications are treated by surgical removal^6^ (Fig. 1).

The most frequent differential diagnoses of PT are lymph node calcifications, foreign bodies, phleboliths, calcified granulomas, calcification of the stylohyoid ligament, calcified triticeous cartilage, submandibular sialoliths and deep fungal infections^10,11^. There are a number of phenomena that should be considered when panoramic radiographs are used, such as the superimposition of anatomical structures, image distortion or even the presence of ghost images. The use of Computed Tomography (CT) offers greater precision, as does the Cone Beam Computed Tomography (CBCT)^12^.

The Dominican Republic is one of the poorest countries in the world, and the various races found therein are clearly differentiated by its citizens. Socially speaking, there are three “races”: black, white (Caucasian) and *mulato* (mixed race). As a rule, this so-called “social race” is associated with level of oral health and social status^13^. Also, social status is frequently associated with the primary medical attention sought (i.e. public health, private assistance and health insurance)^14^. We located a number of studies on PT prevalence in Asian patients^10,12,15,16^ and two studies focusing on Caucasian patients^8,17^, but found no studies that spotlighted black patients specifically. The purpose of this study is to estimate the prevalence of palatine tonsilloliths in a Dominican population sample and to analyze any links with the race of patients.

## Methods

In this cross-sectional study, 209 consecutive patients attending the dental services of two clinics belonging to the Federico Henríquez y Carvajal University (one located in Santo Domingo and the other next to Santiago de los Caballeros, Dominican Republic), from March 1 to April 30, 2019, were selected. All patients (or parent/guardian if the patient was under 18) signed an informed consent before undergoing clinical examination. The study was approved by the Ethics Committees of the health centers where it was carried out.

Computed tomography (CT) images were obtained from scanning patients using a LightSpeed® VCT scanner (General Electric Healthcare, Barrington, IL). The scan field of view and resolution were 320 mm and 512 × 512 pixels, respectively. The slice thickness was 3 mm and the slice spacing or interval was between 1.0 mm and 1.8 mm. Four patients were taken out of the study due to the poor quality of their CT images. For the remaining 205 individuals, relevant data for the purpose of this study was collected using a questionnaire. (Table 1) Mean age of patients (123 females and 82 males, 60% and 40%, respectively) was 40.2 ± 20.53, and they ranged in age from 5 to 93 years.

**Table 1:** Patients’ data collected from questionnaires and evaluation of CT scans.

Sex	Race	Health assistance	City	Previous tonsillitis	Presence of tonsilloliths
*Female*	Mixed race	*Private*	*Sto. Domingo*	*Yes*	*Yes*
123	92	70	105	68	12
*Male*	White	*Public*	*Santiago*	*No*	*No*
82	66	86	100	137	193
	Black	*Insurance*			
	47	49			

CT images were taken in the axial plane, using the inferior orbital rim as the upper limit and the lower edge of the hyoid bone as the lower limit. Standard bone and soft tissue algorithms were used to obtain the examined images. To reduce the risk of bias, all 205 CT images were analyzed separately by two dental radiologists (J. E. and E. A.) who were qualified to read orofacial CT scans and did not participate in taking the CT images. These experts evaluated the images to determine the presence or absence of PT. Radiopaque nodular mass(es) located in the palatine tonsil area were evaluated as presence of PT. Interobserver agreement was 100% for the diagnosis of palatine tonsillar calcifications (κ = 1.00).

Besides age and sex, all Table 1 individuals’ data was registered. The city of residence was recorded according to the medical assistance center, and every patient was racially categorized into one of three groups: mixed race, white or black. Any occurrence of previous tonsillitis was checked, and participants were categorized according to the type of health assistance used, as per one of three groups: private, public or insurance. This information was obtained from the questionnaires and the presence of PT was attained from the radiology experts’ analysis.

Relevant pairs of grouped variables were selected for comparison. A statistical significance level of 95% was considered (α = 0.05) when comparing these pairs. Comparison was carried out using Z-test in SPSS v. 25 (IBM® SPSS® Statistics, Armonk, NY) to compare two proportions. A significant statistical difference was considered between the compared proportions when p-value < 0.05. Otherwise, a null hypothesis was accepted, meaning that the difference between proportions was not significant. Also, a correlation matrix was obtained using the Pearson correlation coefficient for all pairs of variables included in Table 2, in addition to the presence of PT. This analysis was carried out using version 10 of STATISTICA® software (StatSoft, Inc. Tulsa, OK, USA). Significant correlations were considered at p < 0.05.

**Table 2:** Detailed information of the 12 patients diagnosed with PT.

Sex	Age	Race	Health assistance	City	Previous tonsillitis
F	45	MR	Pv	SD	Yes
M	45	W	Pv	SD	No
M	26	MR	Pb	STG	Yes
F	14	W	Pb	STG	Yes
F	34	MR	Pb	STG	Yes
F	19	B	Pb	STG	Yes
F	20	B	Pb	STG	Yes
F	20	W	Pb	STG	Yes
M	41	W	Pv	STG	Yes
F	47	MR	Pv	STG	Yes
M	15	B	Pb	SD	Yes
F	24	MR	Pb	SD	Yes

F: Female; M: Male; MR: Mixed Race; W: White; B: Black; Pv: Private health assistance; Pb: Public health assistance; SD: Santo Domingo; STG: Santiago.

### Ethics approval and consent to participate

Approval was obtained for this study from Federico Henriquez y Carvajal University´s institutional review board. Written consent was obtained from all participants.

## Results

**Figure 2:**
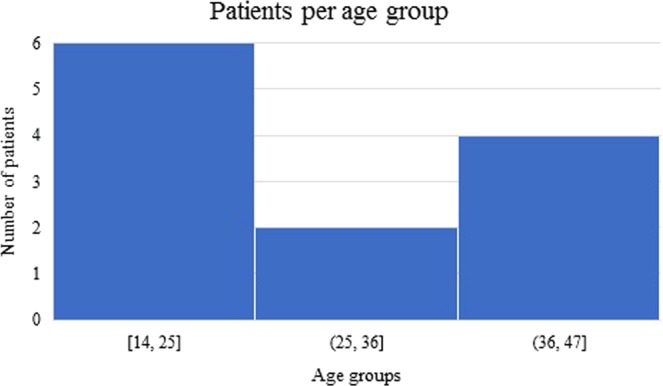
Age groups of patients diagnosed with PT. Half of patients belonged to group A (14–25 years).

Four patients were taken out of the study due to the poor quality of their CT images. PT were found in 12 out of 205 patients’ CT scans (5.85%); four of them were males and the remaining eight were females. The prevalence was 4.88% in males and 6.50% in females with a non-significant difference (p > 0.05). Patients diagnosed with PT ranged from 14 to 47 years of age (Mean = 29.17; SD = 12.51). Seven of those patients were found to have only one calcification (58.33%), while five others presented more than one calcification (41.67%). Three CT scans showed a bilateral occurrence of PT (25.00%). For a better understanding of the results, patients were age-classified into three groups: group A (14–25 years), group B (25–36 years) and group C (36–47 years). Half of the diagnosed patients belonged to group A, two patients were in group B (16.67%) and four patients were in group C (33.33%). (Figure 2) Prevalence in patients under 40 years of age was significantly higher than prevalence in patients over 40 (p < 0.05). (Table 2)

There were significantly more mixed race (92; 44.88%) than white (66; 32.20%) or black (47; 22.93%) patients, and more white patients than black patients (p < 0.05). (Table 1) When the race of patients was considered, mixed race patients received the most diagnoses of PT (five out of twelve, 41.67%). Nevertheless, four (33.33%) white and three (25.00%) black patients were also diagnosed. As such, there were non-significant differences between race groups among diagnosed patients. (Table 2) Also, when grouping mixed race patients (92) and black patients (47) as “black patients,” no significant difference was found between black and white (66) patients with diagnosed PT (8/139 *vs*. 4/66, respectively, p > 0.05). Fourteen black,34 mixed race and 20 white patients reported previous tonsillitis. There was no statistical difference between the three race groups regarding previous tonsillitis. Also, a higher percentage of white patients was found to use private health compared to black patients (p < 0.05), but there was no significant difference between mixed race patients and the other two races. Similarly, there are significantly more black patients in the public health system than white patients (p < 0.05), while no differences between mixed race patients and the other two races were calculated. There was no significant difference between the race groups covered by dental insurance services.

It is remarkable that all patients diagnosed with PT were private or public health patients, and that none of them had dental insurance coverage. (Table 2) Despite this fact, there was no statistical difference between insured patients diagnosed with PT and insured patients in the population sample. The proportion of patients using the public health system in the studied population sample is not significantly different from the proportion of patients using public health among the individuals diagnosed with PT (p > 0.05). Similarly, there was no statistical difference between the percentage of patients using private health in the sample and in the groups of diagnosed patients (p > 0.05). On the other hand, a significantly higher number of male patients utilized private over public health (p < 0.05). In the case of females, the number of private as well as public health patients was significantly greater than dental insurance patients (p < 0.05 in both cases). In females, the proportion of individuals with diagnosed PT utilizing public health is significantly higher than those with dental insurance (p < 0.05). (Table 2)

A significantly greater number of patients reporting previous tonsillitis (68) than patients diagnosed with PT (12) was found (p < 0.05). There were more female (50) than male (18) patients reporting previous tonsillitis (p < 0.05). The Pearson correlation matrix can be seen in Table 3. Sex, presence of PT, previous tonsillitis, city, health assistance, race and age were included in this analysis. The only variable showing a significant (p < 0.05) correlation with presence of PT was reported previous tonsillitis (r = 0.31). Other significant (p < 0.05) correlations can be seen highlighted in Table 3.

**Table 3:** Pearson correlation matrix including all variables. (Significant correlations are marked with*).

	Sex	PT	Previous tonsillitis	City	Health assistance	Race	Age
Sex		0.629	0.005*	0.777	0.031*	0.211	0.219
PT	−0,03		0.000*	0.203	0.277	0.813	0.051
Previous tonsillitis	−0,19*	0,31*		0.522	0.077	0.345	0.005*
City	0,02	−0,09	0,04		0.964	0.605	0.000*
Health assistance	−0,15*	0,08	0,12	0,00		0.872	0.797
Race	−0,09	−0,02	0,07	0,04	0,01		0.141
Age	0,09	−0,14	−0,19*	0,56*	−0,02	−0,10	

P-values are above (or on the right) the diagonal and Pearson correlation coefficient (r) are below (or on the left) the diagonal.

*Significant correlation (p < 0.05).

## Discussion

None of the 205 included patients were previously diagnosed with PT and their visit to the medical center was not related to the presence of these calcifications or associated symptoms. PT prevalence observed in the present study is lower than previously reported. These results may be due to several factors. Regarding race, we had hypothesized some level of correlation but found no significant differences between diagnosed PT and the three studied racial groups. Nevertheless, when comparing our study to previous studies and considering our limitations, a probable influence of race in PT prevalence is suggested.

PT are formed as a result of unsolved tonsillitis; pus cells, and microorganism chemical and biological derivatives (debris) create a base environment for calcification genesis in tonsillar crypts^18^. This calcification may promote the inflammation of surrounding tissue and halitosis; however, none of the diagnosed cases of PT were associated with any present clinical signs. In the present study, a 5.85% value was determined for prevalence of PT in the studied population sample. This value is lower than previously reported, which ranged from 16.0%^17^ to 46.1%^15^ for studies using CT images. (Table 4) Many factors should be taken into consideration when comparing previously reported PT prevalence values. Perhaps the most important might be the CT imaging parameters used. Previous studies have stressed the significantly higher sensitivity of CT methods for the diagnosis of PT, when compared to the use of panoramic radiographies^10,15^. Also, some authors believe that the used slice thickness and the slice spacing (or interval) may strongly influence the determined prevalence, as these parameters could affect the detection process and sensitivity^10,12,15,16^. (Table 4)

**Table 4:** Prevalence results, sample sizes and CT image data from previous studies. Comparison to present study.

Author	Year	Country	PT prevalence	N	Slice thickness	Interval
Aspestrand & Kolbenstvedt^17^	1987	Norway	16.0%	100	5 mm	0–3 mm
Fauroux *et al*.^8^	2013	France	24.6%	150	0.625–1.250 mm	0.2–1.0 mm
Oda *et al*.^15^	2013	Japan	46.1%	482	3 mm	Contiguous
Takahashi *et al*.^16^	2014	Japan	39.9%	2873	1 mm	—
Kajan *et al*.^12^	2016	Iran	40.3%	134	0.5 mm	0.5 mm
Takahashi *et al*.^10^	2017	Japan	40.7%	2244	1 mm	—
Present study	2019	Dominican Republic	5.85%	205	3 mm	1.0–1.8 mm

The number of included cases is yet another factor to be taken into account; statistically speaking, the greater the number, the more reliable the result. Takahashi *et al*.^10,16^ and Kajan *et al*.^12^ reported similar PT prevalence (about 40%), but the Takahashi studies (in Japan) included almost 20 times the number of patients Kajan *et al*.^12^ included (in Iran). Despite the Iranian study using a smaller slice thickness, the reduced number of cases might have affected the prevalence results. The higher prevalence was reported by Oda *et al*.^15^ (46.1%). These authors worked with 482 CT scan images and a 3 mm slice thickness was used. That said, the contiguously used interval may have augmented the method sensitivity. As for Aspestrand & Kolbenstvedt^17^ and Fauroux *et al*.^8^, they used fewer patients, but different CT parameters; the latter used a much less significant slice thickness and interval, and reported a higher PT prevalence (24.6% *vs*. 16%). Considering only these two factors (CT parameters and number of cases), differences between previous studies and our results could be partially explained. We used a greater slice thickness and interval than most of the previous works (except for Aspestrand & Kolbenstvedt^17^). This could lead us to believe that the low determined prevalence may have been due to that. However, Aspestrand & Kolbenstvedt^17^ used a higher slice thickness and interval, and a smaller sample of patients, and reported almost three times our value of prevalence (16.0% *vs*. 5.9%).

As expected, PT prevalence appears to be spurred by multiple factors. In addition to the two previously mentioned (CT parameters and number of cases), we also considered race. Kumagai *et al*.^19^ reported a ten-fold prevalence of carotid artery calcifications in Japanese patients when compared to African Americans (reported by Hubar^20^). In light of this, Takahashi *et al*.^10^ considered that the occurrence of some types of pathological/physiological calcifications might be dependent on (or somehow related to) race. Explicitly considering PT, the reported prevalence was 39.9%, 40.7% and 46.1% for studies conducted in Japan^10,15,16^; 40.3% for the study conducted in Iran;^12^ 24.6% for the French study^8^; and 16.0% for the study conducted in Norway^17^. (Table 4) A clear trend can be inferred from these results: higher PT prevalence among Asian populations than Europeans. Regarding the Iranian study (also a high PT prevalence)^12^, it must be noted that the researchers used the smallest values of slice thickness and interval in CT scan image reconstruction of all the studies under consideration.

We presented a PT prevalence of 5.85% for a Dominican population sample composed mostly of African-Caucasian descendants. If race influences the rate of pathological/physiological calcification formation, both Caucasian and African patients are reported to have lower prevalence than Asian patients, and this could be a reasonably relevant factor biasing our results (in comparison to previous studies). Though we did not find any significant differences between the included races for PT diagnosed patients, the absence of Asian patients in our study might suggest the possibility of a racial factor in our prevalence results (when compared with the results of other authors).

Regarding race and taking into account the observed (in previous publications) differences between Asian and Caucasian patients concerning PT prevalence, we could only compare our results to the two Caucasian-based studies^8,17^. (as our study lacks Asian patients). As CT slice thickness and interval influence detection sensitivity, when considering the used PT detection method parameters, the Aspestrand & Kolbenstvedt^17^ publication is most similar to ours. So, logically, we decided to use their results for comparison purposes. In this sense, there were no significant differences in PT prevalence among Caucasian patients for both studies (p > 0.05). And there were more Caucasian patients with diagnosed PT in Aspestrand & Kolbenstvedt’s study^17^ (16/100) than black patients with diagnosed PT (8/139) in our study (p < 0.05). These findings could support the results reported by Kumagai *et al*.^19^, Hubar^20^ and Takahashi *et al*.^10^ regarding the influence of race on the prevalence of calcifications.

The majority of Dominicans are mixed race. That being said, a number of white patients are tourists or temporary workers, while black patients are generally Haitian descendants. One must take into consideration that the Dominican population is highly mixed, racially speaking. Many of the native “white” patients have Caucasian and African origins, although their skin is significantly lighter than that of mixed race patients. As a rule, the social status and income of Dominicans is related to race. In this sense, we found that a higher percentage of white patients used private health when compared to black patients (p < 0.05). Also, there are significantly more black patients in the public health system than white patients (p < 0.05). These findings are in accordance with the social role played by race in the Dominican population.

Regarding the kind of health assistance used, some trends were found. First, men are apparently more likely to utilize private health than public health or have dental insurance. Meanwhile, women are more likely to use the public and private health systems, than be covered by dental insurance. Dominican society is highly sexist and, as such, women are more likely to use the public health system than men (as well as have lower salaries and worse jobs).

Some authors previously reported correlations between PT prevalence and age of participants^10,15,16^. Their results (all three are based on Japanese studies) suggest that there are highly significant differences between PT prevalence in patients over 40 years and younger patients. These authors believe this might be caused by higher rates of smoking and/or poor oral hygiene. Our findings suggest the opposite. There was no statistical difference between the three age groups defined in Fig. 2, regarding the population sample included in this study. Nonetheless, when considering patients diagnosed with PT, 6 (50.0%) of the patients were in the 14–25 age group, while only 4 (33.3%) were over 40. This discrepancy with previous studies may be due to differences in race or hygiene habits associated with the culture of the population sample.

The only variable showing a significant (p < 0.05) correlation with presence of PT was reported previous tonsillitis (r = 0.31). (Table 3) As a matter of fact, eleven out of twelve patients diagnosed with PT had had tonsillitis. These results suggest a relationship between PT and previous tonsillitis, in accordance with earlier studies and the presumed PT link with tonsillar infections and oral hygiene.

Taking into account the discussed probable association between race and PT prevalence, together with racial features of the Dominican population, the observed results could probably be applied to populations with similar characteristics regarding race. This kind of population is commonly found in the Caribbean area. Countries whose populations are majority African descendants could also be considered.

## Conclusions

Considering the limitations of this study (sample size, diagnostic methodology, etc.), the present results suggest that PT prevalence among patients seeking treatment at two university clinics in the Dominican Republic is relatively lower (5.85%) than previously reported in the populations of other countries. This difference may be due to several factors, such as diagnostic method parameters, sample size or racial characteristics. Racial differences may influence the kind of healthcare sought (private, public or insurance). More white patients use private health while more black patients use the public system. Also, we found no significant relationship between PT prevalence and race. Despite this, when comparing our study to a previous similar study and considering the Asian-centric results obtained, a race influence on prevalence of calcifications might be suggested. In this sense, black patients appear to be less likely to present symptoms of PT than Caucasian and Asian patients. A significant correlation was found between PT prevalence and previous tonsillitis. According to the obtained results, young patients (14–25 years) are more likely to have PT than older patients in the studied population, in contrast with previous studies in other countries.

## Data Availability

The data and material are available from the corresponding author.
